# Dysbiosis of Bile Microbiota in Cholangiocarcinoma Patients: A Comparison with Benign Biliary Diseases

**DOI:** 10.3390/ijms26041577

**Published:** 2025-02-13

**Authors:** Wonsuk Park, Sang Kuon Lee, Jin Gwack, Seung Yeob Lee, Yong Gon Cho, Sang-Bum Kang, Joonhong Park

**Affiliations:** 1 Division of Gastroenterology, Department of Internal Medicine, Daejeon St. Mary’s Hospital, College of Medicine, The Catholic University of Korea, Seoul 06591, Republic of Korea; mdonekr@naver.com; 2 Department of Surgery, College of Medicine, The Catholic University of Korea, Seoul 06591, Republic of Korea; luislee@catholic.ac.kr; 3 Department of Prevention Medicine, College of Medicine, Jeonbuk National University, Jeonju 54907, Republic of Korea; gwackjin@jbnu.ac.kr; 4 Research Institute of Clinical Medicine of Jeonbuk National University-Biomedical Research Institute of Jeonbuk National University Hospital, Jeonju 54907, Republic of Korea; seungyeoblee@jbnu.ac.kr (S.Y.L.); choyg@jbnu.ac.kr (Y.G.C.); 5 Department of Laboratory Medicine, Jeonbuk National University College of Medicine and Hospital, Jeonju 54907, Republic of Korea

**Keywords:** bile microbiota, dysbiosis, cholangiocarcinoma, next-generation sequencing, 16S rRNA gene, EzBioCloud

## Abstract

Dysbiosis in the bile microbiota of cholangiocarcinoma (CCA) patients suggests a potential role for microbial alterations in the pathogenesis of CCA. This study aimed to investigate bile microbial communities in patients with CCA and compare them to those in individuals with benign biliary diseases as a control (CTR) group. Microbial profiling was conducted using next-generation sequencing (NGS), targeting the V3–V4 regions of the 16S rRNA gene, followed by bioinformatics analysis using the VSEARCH and EzBioCloud platforms. Alpha and beta diversity analyses were performed to assess microbial richness and structural differences. The linear discriminant analysis effect size (LEfSe) was utilized to identify potential microbial biomarkers. Results: This study identified distinct microbial profiles in the two groups at both the phylum and genus levels. In the CTR group, *Pseudomonadota* (65%) was the dominant phyla, while *Bacillota* (49%) was more abundant in the CCA group. At the genus level, *Escherichia* (29%), *Enterobacteriaceae* (12%), *Enterococcus* (8%), *Ralstonia* (8%), and *Clostridium* (5%) were more prevalent in the CTR group, whereas *Streptococcus* (34%), *Ralstonia* (8%), and *Veillonella* (5%) were dominant in the CCA group. Although an alpha diversity analysis showed no statistically significant differences in species richness or diversity between groups, a beta diversity analysis revealed significant structural differences associated with disease severity. Our comparative microbiome study using LEfSe analysis suggested a statistically significant inhibition of normal intestinal bacterial flora in patients with CCA who had not received any treatment. These findings suggest that microbial dysbiosis may play a role in the pathogenesis of CCA. Specific microbial taxa were identified as potential biomarkers for distinguishing benign from malignant diseases. These results underscore the potential role of microbial dysbiosis in CCA pathogenesis and highlight the bile microbiota’s utility as a diagnostic marker for biliary diseases.

## 1. Introduction

Cholangiocarcinoma (CCA), a malignant tumor of the biliary system, is the second most common primary hepatobiliary cancer, comprising approximately 15% of cases in this category [1,2]. Despite its relatively low overall prevalence, CCA represents 3% of all digestive cancers and has a poor prognosis, with a 5-year survival rate of just 5%. Its diagnosis is frequently delayed due to the absence of specific symptoms in the early stages, resulting in about 70% of cases being identified only at advanced stages [3]. CCA is further categorized, based on its anatomical location, into intrahepatic, perihilar, and distal types [4]. Intrahepatic CCA is the second most common primary liver cancer after hepatocellular carcinoma, accounting for 10–20% of cases. Globally, the incidence of CCA is rising, particularly in certain Asian countries rather than Western regions. While diagnostic methods like endoscopic retrograde cholangiopancreatography (ERCP) are invasive, they remain critical for both its diagnosis and therapeutic management [5].

Cholestasis and chronic inflammation are key mechanisms driving CCA tumorigenesis. Conjugated bile acids play a pivotal role by promoting the secretion of growth factors and suppressing apoptosis, ultimately leading to proto-oncogene activation and the malignant transformation of bile duct cells [6,7]. Several independent risk factors have been identified for CCA, including primary sclerosing cholangitis, hepatobiliary parasites, bile duct stones, choledochal cysts, and exposure to carcinogens such as asbestos and nitrosamines. Additionally, liver cirrhosis, hepatitis B and C infections, obesity, diabetes, smoking, alcohol consumption, and enteric circulation disorders are recognized as contributing factors [4,8]. However, the majority of patients do not exhibit identifiable risk factors, leaving the etiology and pathogenesis of CCA largely unknown. Recent evidence highlights the potential role of genetic and epigenetic alterations influenced by the human microbiome in CCA development [9,10]. Traditionally, bile—composed mainly of bile acids, cholesterol, and phospholipids—was believed to be sterile due to its antimicrobial properties. However, metagenomic studies have revealed the presence of non-cultivable bacteria in the gallbladder under non-pathological conditions [11,12]. Recent metagenomic studies have demonstrated that bile is not a sterile environment, even in the absence of pathological conditions, as specific microbial communities have been identified in healthy individuals. However, research on the biliary microbiome in healthy individuals remains limited, making it difficult to establish a well-defined baseline microbial profile. In their study, Molinero et al. [13] provided valuable insights into the microbial composition of the human gallbladder in individuals without hepatobiliary diseases, reporting that the predominant bacterial phyla include *Bacillota*, *Bacteroidota*, *Actinomycetota*, and *Pseudomonadota*. Notably, these microbial compositions can undergo significant shifts during disease progression, which have been associated with various hepatobiliary and gastrointestinal disorders, potentially influencing bile acid metabolism and gut microbiota composition [13,14]. The human microbiome, which encompasses microbial communities in the intestines, oral cavity, skin, nasal passages, and vaginal tract, plays a crucial role in digestion, immunity, and host metabolism [15]. While 16S ribosomal RNA sequencing has enabled taxonomic profiling from the phylum to genus level, achieving species-level resolution often necessitates whole-metagenome sequencing. This highlights the importance of further research to better understand the contribution of the bile microbiome to both health and disease.

Recent studies have revealed significant differences in the composition of the bile microbiota between patients with CCA and healthy individuals, suggesting that microbial communities in the biliary tract may play a critical role in the development and progression of CCA. Research has identified an enrichment of specific bacterial genera, such as *Enterococcus, Streptococcus, Bacteroides, Klebsiella,* and *Pyramidobacter,* in the bile of CCA patients [16]. At the same time, a reduction in microbial diversity has been observed in these patients, potentially creating conditions conducive to carcinogenesis [17]. This microbial dysbiosis, characterized by both compositional shifts and decreased diversity, may contribute to CCA pathogenesis through mechanisms such as chronic inflammation induced by particular bacterial species and the production of carcinogenic metabolites. These processes can promote the malignant transformation of biliary epithelial cells, emphasizing the possible link between microbial imbalances and cancer development. The findings highlight the importance of further research in understanding the role of the bile microbiota in CCA and its potential as a target for therapeutic interventions [17].

The primary objective of this study was to examine the bile microbial communities in patients with CCA and compare them to those in individuals with benign biliary diseases. Given the ethical and practical challenges of collecting bile from healthy individuals, patients with benign biliary diseases were selected as a control (CTR) group to provide a more feasible and clinically relevant comparison. By leveraging next-generation sequencing (NGS) technology and advanced bioinformatics tools, this research aimed to achieve several goals: to characterize the composition of the bile microbiota, evaluate microbial diversity, identify potential biomarkers, and explore the associations between microbial profiles and disease severity.

## 2. Results

This study included 25 patients, of whom 10 (40%) were male. The median age of the participants was 58 years, with an age range of 32 to 84 years. In the CTR group, 12 individuals had gallstones confined to the common bile duct (CBD) or gallbladder, while 3 had gallbladder polyps. In contrast, all 10 participants in the CCA group were diagnosed exclusively with CCA in the CBD. No significant differences in age or sex were observed between the two groups.

### 2.1. Proportional Taxonomic Composition of Bile Microbial Communities

**Figure 1:**
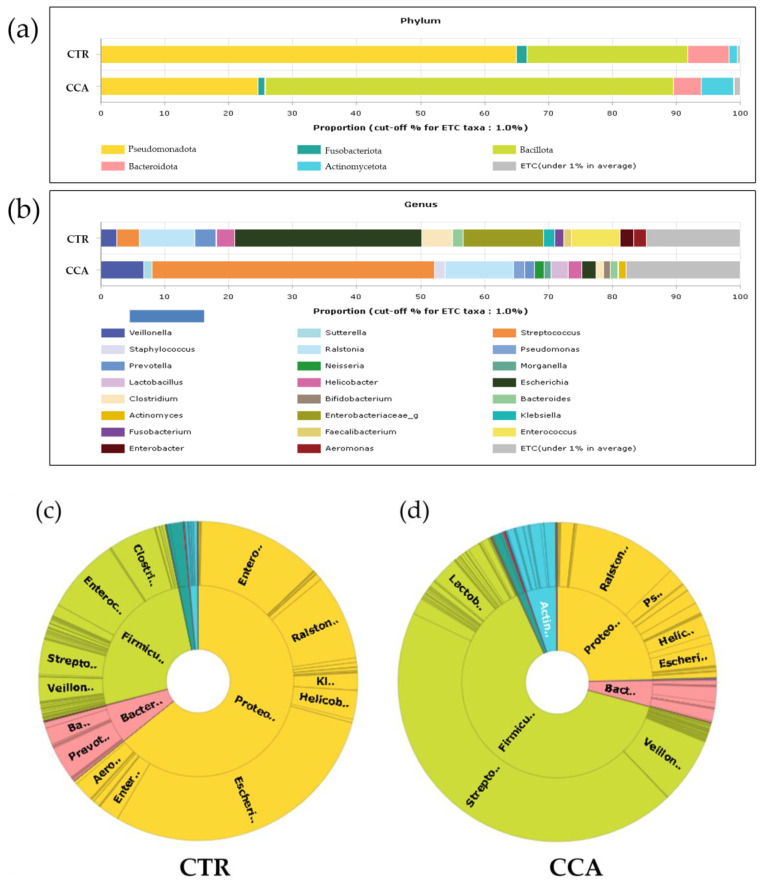
The taxonomic composition of bile microbial communities at the phylum and genus levels was analyzed and compared between the benign biliary diseases as a control (CTR) group and the cholangiocarcinoma (CCA) group. Stacked bar charts illustrate the taxonomic composition at the phylum level (**a**) and genus level (**b**). The term “ETC” (et cetera) represents the population of identified phyla or genera present at a proportion of less than 1%. At the genus level, the taxonomic profiling reveals that 1% of the composition of the higher taxonomic ranks remains unassigned, whereas no unassigned taxa were identified at the phylum level. The x-axis in the charts represents the relative abundance as a percentage. Double pie charts further depict the taxonomic compositions at the phylum level (inner circle) and genus level (outer circle) of the CTR group (**c**) and the CCA group (**d**).

The analysis of the microbial communities in the CTR and CCA groups revealed five dominant phyla. In the CTR group, these phyla were identified in the following order of prevalence: *Pseudomonadota* (65%), *Bacillota* (25%), *Bacteroidota* (6%), *Fusobacteriota* (2%), and *Actinomycetota* (1%). Conversely, in the CCA group, *Bacillota* (49%) was the most dominant, followed by *Pseudomonadota* (40%), *Actinomycetota* (4%), and *Bacteroidota* (3%) (Figure 1a). Significant differences in the relative abundance and distribution of these phyla were observed between the two groups. At the genus level, 15 prevalent genera were identified in the CTR group, compared to 13 genera in the CCA group. The five most prevalent genera in the CTR group were *Escherichia* (29%), *Enterobacteriaceae* (12%), *Ralstonia* (8%), *Enterococcus* (8%), and *Clostridium* (5%). While most of these genera were predominantly found in the CTR group, *Ralstonia* was also identified in the CCA group. In contrast, the CCA group was dominated by *Streptococcus* (34%), *Ralstonia* (8%), and *Veillonella* (5%) (Figure 1b). Comparative analysis using double pie charts highlighted the distinct differences in the proportions of the bacterial taxa between the groups. Notably, the proportion of *Pseudomonadota* was significantly higher in the CTR group, driven by the presence of *Escherichia* and *Enterobacteriaceae*, which appeared in this group alone. However, the overall composition of *Pseudomonadota* did not significantly differ between the groups (Figure 1c). In contrast, *Bacillota*, including genera such as *Streptococcus, Ralstonia,* and *Lactobacillus*, were more abundant in the CCA group (Figure 1d).

**Figure 2:**
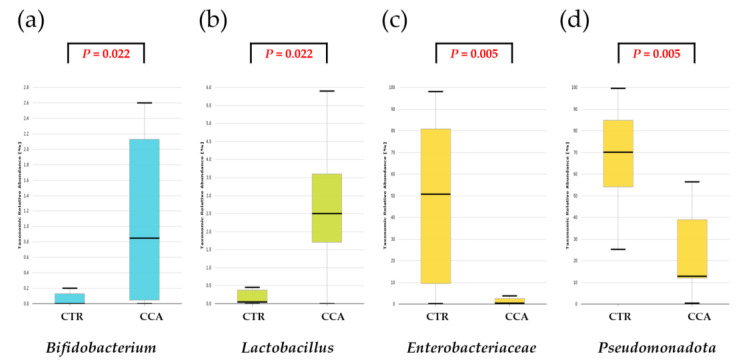
A comparative analysis was conducted on the taxonomic composition of four selected taxa known for their significance in the human gastrointestinal tract. The benign biliary diseases as a control (CTR) group, consisting of patients with gallstones or gallbladder polyps, exhibited a lower relative abundance of *Bifidobacterium* (**a**) and *Lactobacillus* (**b**) compared to the cholangiocarcinoma (CCA) group. Conversely, the CTR group demonstrated a higher relative abundance of *Enterobacteriaceae* (**c**) and *Pseudomonadota* (**d**) relative to the CCA group.

A further comparative analysis of specific taxa—*Bifidobacterium, Lactobacillus, Enterobacteriaceae*, and *Pseudomonadota*—known for their relevance to the human gut microbiota revealed significant differences in their relative abundance between the CTR and CCA groups. The CCA group showed significantly higher levels of *Bifidobacterium* (*p* = 0.022) and *Lactobacillus* (*p* = 0.022), while the CTR group exhibited significantly higher levels of *Enterobacteriaceae* (*p* = 0.005) and *Pseudomonadota* (*p* = 0.005) (Figure 2).

### 2.2. Analysis of Alpha Diversity in Bile Microbial Communities

**Figure 3:**
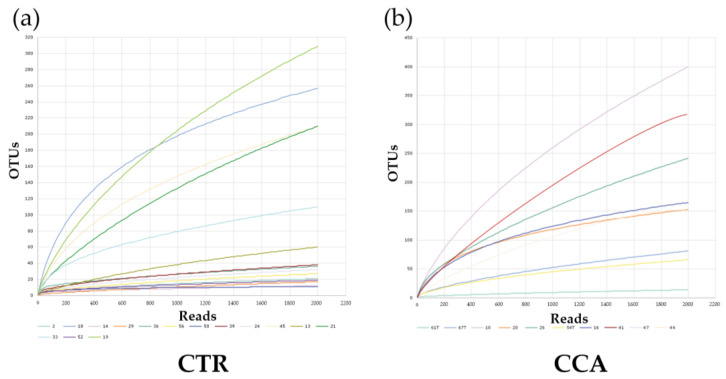

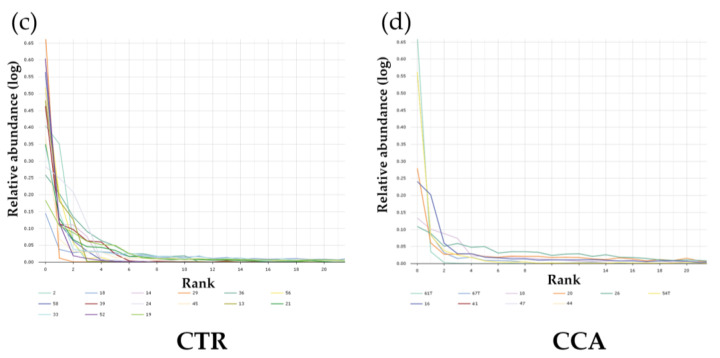
The rarefaction and rank abundance curves for the benign biliary diseases as a control (CTR) group, consisting of patients with gallstones or gallbladder polyps, and the cholangiocarcinoma (CCA) group were visualized to assess microbial diversity and distribution. Rarefaction curves and species richness indices indicate the extent of our comprehensive sampling in the CTR group (**a**) and the CCA group (**b**). A broader span in the rank abundance curves reflects higher relative species abundance, while a smoother curve along the Y-axis signifies greater evenness in bacterial distribution for the CTR group (**c**) and the CCA group (**d**).

Species richness and diversity in the CTR and CCA groups were assessed through the generation of rarefaction curves based on sequence counts from each sample. The rarefaction curves for both groups plateaued, as observed in the CTR group (Figure 3a) and the CCA group (Figure 3b), indicating that a sequencing depth of 2000 was sufficient to accurately estimate species richness and diversity within each sample. Rank abundance curves were also utilized to compare the microbial community structures of the two groups. The distinct patterns in the curves for the CTR group (Figure 3c) and the CCA group (Figure 3d) suggest notable differences in their microbial community structures.

**Figure 4:**
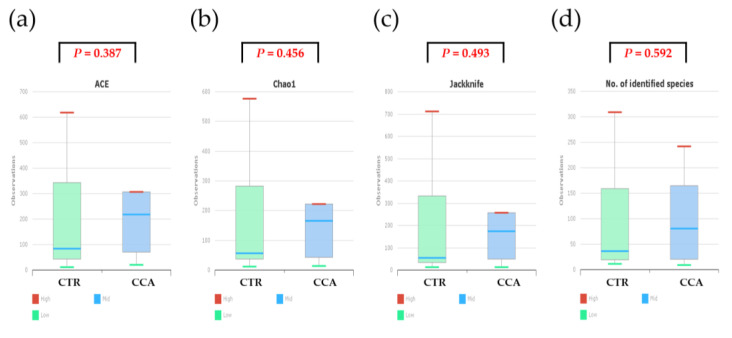

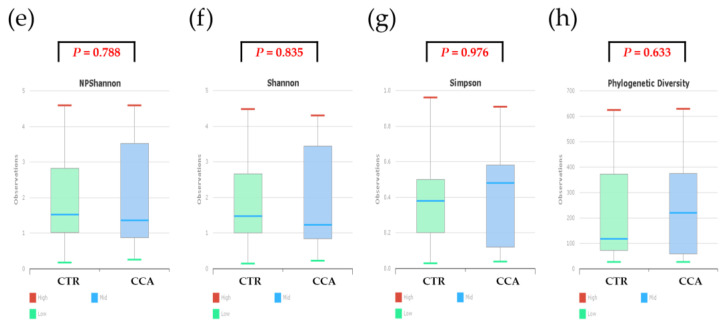
The alpha diversity of bile microbial communities at the genus level was analyzed for the benign biliary diseases as a control (CTR) group, consisting of patients with gallstones or gallbladder polyps, and the cholangiocarcinoma (CCA) group using massively parallel 16S rRNA gene sequencing. Alpha diversity was evaluated in terms of species richness (**a**–**d**) and diversity indices (**e**–**h**) within bile microbial communities collected from the common bile duct in the CTR group and the gallbladder in the CCA group. The red lines indicate high values, the blue lines represent median values, and the green lines show low values.

Alpha diversity was analyzed using indices such as Jackknife, Chao1, and ACE to evaluate variations in the bile microbial communities of the two groups. The analysis revealed that the bacterial diversity in bile samples was lower in the CTR group compared to the CCA group, although the differences were not statistically significant (Figure 4a–c). When normalized to a consistent read count based on gene copy numbers, alpha diversity, as measured by species richness, remained lower in the CTR group than in the CCA group, but the difference was not statistically significant (Wilcoxon rank-sum test, *p* = 0.592; Figure 4d). Further analysis of alpha diversity indices, including NPShannon (*p* = 0.788), Shannon (*p* = 0.835), Simpson (*p* = 0.976), and phylogenetic diversity (*p* = 0.633), also showed no statistically significant differences between the two groups (Figure 4e–h).

### 2.3. Analysis of Beta Diversity in Bile Microbial Communities

**Figure 5:**
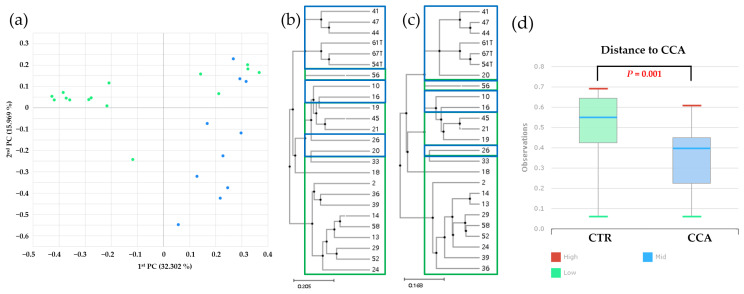
Beta diversity within bile microbial communities at the genus level was assessed for the benign biliary diseases as a control (CTR) group, consisting of patients with gallstones or gallbladder polyps, and the cholangiocarcinoma (CCA) group using massively parallel 16S rRNA gene sequencing. (**a**) A principal coordinate analysis (PCoA) revealed distinct clustering patterns, indicating differences in overall bile microbial community composition between the CTR group (solid green dots) and the CCA group (solid blue dots) based on gallstone location. A hierarchical clustering analysis, performed using the unweighted pair group method with arithmetic mean (UPGMA), demonstrated variations in microbial abundance and diversity between the CTR group (empty green boxes) and the CCA group (empty blue boxes), based on generalized UniFrac (**b**) and UniFrac (**c**) metrics. (**d**) Differences in diversity indices between the CTR and CCA groups were quantified and presented in representative box plots using permutational multivariate analysis of variance (PERMANOVA). The red lines indicate high values, the blue lines represent median values, and the green lines signify low values.

Beta diversity analysis was performed to investigate the bile microbial communities at the genus level in the CTR and CCA groups, utilizing massively parallel 16S rRNA gene sequencing. The analysis included a principal coordinate analysis (PCoA) based on generalized UniFrac and UniFrac metrics. The PCoA results demonstrated distinct clustering patterns between the CTR group (solid green dots) and the CCA group (solid blue dots), reflecting differences in disease severity, with the CTR group representing benign diseases and the CCA group representing malignancy (Figure 5a). To further explore abundance and diversity differences, a hierarchical clustering analysis was conducted using the unweighted pair group method with arithmetic mean (UPGMA). This analysis, based on generalized UniFrac (Figure 5b) and UniFrac (Figure 5c) metrics, revealed clear distinctions between the CTR group (empty green boxes) and the CCA group (empty blue boxes). A quantitative assessment of diversity differences between the groups was performed using permutational multivariate analysis of variance (PERMANOVA), and the results were visualized as representative box plots, highlighting significant differences in their bile microbial communities (Figure 5d). These findings underscore the substantial differences in the bile microbial communities between patients with benign and malignant diseases, suggesting a potential relationship between microbial composition and disease severity.

### 2.4. Identification of Taxonomic Biomarkers in Bile Microbial Communities

**Figure 6:**
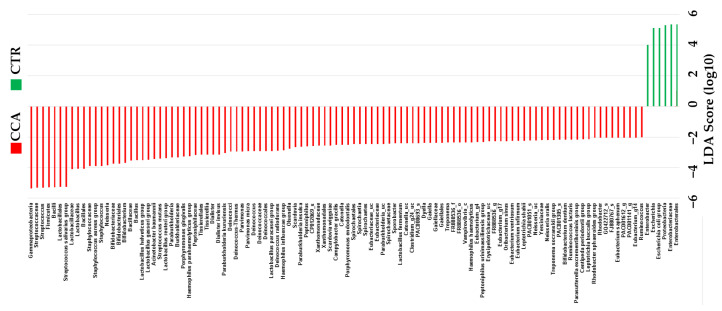
The taxonomic distribution within the benign biliary diseases as a control (CTR) group, consisting of patients with gallstones or gallbladder polyps, and the cholangiocarcinoma (CCA) group was analyzed using the linear discriminant analysis effect size (LEfSe). The analysis was performed with a linear discriminant analysis (LDA) score threshold > 3 and the Kruskal–Wallis test set at a significance level of 0.05.

The linear discriminant analysis effect size (LEfSe) was utilized to identify taxonomic biomarkers with significant differences between the CTR and CCA groups. The analysis applied a linear discriminant analysis (LDA) score threshold of >3 and used the Kruskal–Wallis test with a significance level of α < 0.05. Several genera were identified as potential biomarkers, with the most prominent distinctions observed for *Enterobacteriaceae* (LDA score of 5.34), *Pseudomonadota* (LDA score of 5.31), *Escherichia* (LDA score of 5.12), and *Enterobacter* (LDA score of 4.01). Using the LEfSe analysis, our comparative microbiome study indicated alterations in the intestinal bacterial flora of CCA patients who had not undergone treatment. These results indicate that these specific taxa may serve as biomarkers for bile microbial communities, providing us with a means to differentiate between benign and malignant diseases (Figure 6).

## 3. Discussion

The human microbiome, encompassing microbial communities in the gut, bile, bile duct tissue, and blood, plays a critical role in the tumorigenesis and progression of CCA. A study by Jia et al. [18], involving 84 participants (12 healthy controls, 16 participants with liver cirrhosis, 28 with hepatocellular carcinoma (HCC), and 28 with intrahepatic CCA), highlighted the dominance of phyla such as *Verrucomicrobiota*, *Actinomycetota*, *Bacteroidota*, and *Bacillota* in the gut microbiome. The study also found that patients with intrahepatic CCA exhibited the highest gut microbiome diversity, as measured by alpha diversity and beta diversity indices. At the genus level, *Lactobacillus, Actinomyces, Peptostreptococcaceae,* and *Alloscardovia* were significantly enriched in the intrahepatic CCA group, while *Leuconostocaceae* and *Ruminococcus* were more prevalent in healthy controls. The study further identified associations between specific bacterial genera and clinical parameters. For instance, *Lactobacillus* and *Alloscardovia* were positively correlated with plasma levels of tauroursodeoxycholic acid, whereas the genus *Pseudoramibacter* was negatively associated with these levels and with patient survival time. Notably, tauroursodeoxycholic acid was positively linked to vascular invasion. Additionally, plasma tricarboxylic acid and interleukin-4 showed positive correlations with vascular invasion, suggesting their involvement in disease progression [18]. Microbial dysbiosis has been identified as a key factor in the progression of CCA and its associated precancerous and cancerous conditions [19]. The gut and bile microbiomes influence disease mechanisms, including antitumor immunity, inflammation, and metabolic changes [20]. Mouse models of CCA have provided further insights into these mechanisms. In these models, intestinal barrier disruption allows intestinal bacteria and lipopolysaccharides to translocate to the liver via the portal vein. This process activates a TLR4-dependent pathway and the CXCL1-CXCR2 axis, leading to the accumulation of polymorphonuclear myeloid-derived suppressor cells (PMN-MDSCs), which facilitate immune evasion and promote tumor progression. Interestingly, altering the structure of the gut microbiome in healthy mice through the oral administration of neomycin partially depleted the microbiome and inhibited tumor growth. This effect was attributed to decreased *CXCL1* expression and the reduced aggregation of PMN-MDSCs [21]. These findings underscore the significant role of gut microbial dysbiosis in CCA progression and suggest potential therapeutic targets.

Several studies have highlighted significant differences in the bile microbiome of patients with CCA compared to those with gallstones. In one study, bile samples from patients with perihilar CCA and gallstones were collected and analyzed. The results revealed that *Enterococcus, Bacteroides, Klebsiella,* and *Pyramidobacter* were the most dominant bacterial genera in the bile of perihilar CCA patients. Additionally, patients with perihilar CCA exhibited an enrichment of the phyla *Nitrospirota* and *Gemmatimonadota*, as well as the genera *Geobacillus* and *Bacteroides,* compared to those with gallstones [16,22]. A separate study conducted by Bednarsch et al. [23] investigated bacterial colonization in the bile of PCC patients using bacterial culture techniques. They identified extensive colonization, with *Escherichia coli, Enterobacter cloacae, Enterococcus faecium,* and *Enterococcus faecalis* being the most frequently detected bacterial species. Despite these findings, it remains uncertain whether the distinct bacterial profiles observed in biliary fluid are directly associated with biliary carcinogenesis. Further research is needed to explore the potential relationship between biliary dysbiosis and the development of biliary tract malignancies. Environmental and genetic factors have also been implicated in the pathogenesis of CCA [24]. In a recent study [16], the biliary microbiota of CCA patients was characterized by lower levels of *Bacillota* and higher levels of *Bacteroidota* compared to controls. These studies identified *Enterococcus*, *Streptococcus*, *Bacteroides*, *Klebsiella*, and *Pyramidobacter* as the most abundant genera in the biliary microbiota of CCA patients, with *Bacteroides*, *Geobacillus*, *Meiothermus*, and *Anoxybacillus* being significantly more prevalent in CCA patients compared to controls without associated diseases. However, our findings present a contrasting profile. In our CTR group, the dominant phylum was *Pseudomonadota*, while *Bacillota* was more abundant in the CCA group. At the genus level, *Escherichia*, *Enterobacteriaceae*, *Enterococcus*, and *Clostridium* were dominant in the CTR group, whereas *Streptococcus* and *Veillonella* were dominant in the CCA group. Furthermore, specific taxa, including *Enterobacteriaceae*, *Escherichia*, and *Enterobacter* show potential as biomarkers for differentiating between benign and malignant biliary diseases. These findings suggest a potential role for biliary dysbiosis in CCA development, necessitating further investigation.

Our study employed a standardized and rigorous methodology for bile sample collection, ensuring high sample integrity and minimizing contamination. Sterile techniques were implemented during procedures such as ERCP and laparoscopic cholecystectomy (LC), enhancing the reliability of the findings. To comprehensively and accurately analyze microbial communities, NGS was utilized, targeting the V3–V4 regions of the 16S rRNA gene. Advanced bioinformatics tools, including VSEARCH and EzBioCloud, were applied to enhance the precision of our microbial identification and taxonomic profiling, enabling robust conclusions regarding microbial diversity and abundance. The inclusion of both benign and malignant biliary diseases provided a robust comparative framework for exploring the relationship between microbial composition and disease states. Distinct microbial profiles were identified at both the phylum and genus levels. In the CTR group, *Pseudomonadota* was predominant, whereas the CCA group showed a higher abundance of *Bacillota*. At the genus level, *Escherichia* and *Enterobacteriaceae* were significantly enriched in the CTR group, while *Streptococcus* was more prevalent in the CCA group. A dual approach was employed to analyze alpha and beta diversity, providing detailed insights into microbial richness and structural differences between the groups. The alpha diversity analysis, utilizing indices such as ACE, Chao1, Shannon, and Simpson, revealed no statistically significant differences in overall diversity or species richness between the groups. However, the beta diversity analysis, conducted through PCoA and hierarchical clustering methods such as UPGMA, demonstrated significant structural variations in the bile microbiota between the CTR and CCA groups. Our comparative microbiome study using LEfSe analysis suggested a statistically significant inhibition of normal intestinal bacterial flora in patients with CCA who had not received any treatment. These findings suggest a potential link between microbial community structure and disease severity. Specific microbial taxa, including *Enterobacteriaceae*, *Pseudomonadota*, *Escherichia*, and *Enterobacter,* were identified as potential biomarkers for distinguishing benign from malignant diseases through LEfSe analysis. To interpret various bacteria with a negative LDA score as potential biomarkers for CCA, further studies are needed to elucidate common or consistent pathophysiological mechanisms and characteristics among these bacteria. As these additional experiments were not conducted in our study, this hypothesis remains unsupported. These results have significant clinical implications, offering potential tools for the early diagnosis and prognosis of CCA. The observed structural differences in microbial communities between the groups further support the hypothesis that certain microbial taxa may influence disease progression and a patient’s response to treatment. This study underscores the potential involvement of bile microbiota in the pathogenesis of CCA, suggesting that microbial dysbiosis may contribute to disease development. The identified biomarkers present promising opportunities for the development of diagnostic tools to aid in the early detection of biliary malignancies and facilitate targeted therapeutic interventions.

The diagnostic potential of the human microbiome has been extensively demonstrated across various diseases, including colorectal cancer [25], liver cirrhosis [26], autoimmune hepatitis [27], and type 2 diabetes [28]. For CCA specifically, Jia et al. [18] developed a non-invasive diagnostic model using logistic regression. This model incorporated two microbial genera, *Lactobacillus* and *Alloscardovia*, and achieved impressive area under the curve values of 0.987, 0.965, and 0.968 for distinguishing CCA from healthy controls, liver cirrhosis, and HCC, respectively. These findings highlight the significant potential of microbiome-based biomarkers for diagnosing CCA. While the gut microbiome has shown promise as a diagnostic tool, further research is necessary to explore the diagnostic value of other microbiomes beyond the gut. Expanding this line of investigation could uncover additional non-invasive biomarkers, ultimately enhancing early detection and improving clinical outcomes for CCA patients.

Our study has several limitations that warrant acknowledgment. First, the small sample size—a total of 25 participants (15 in the CTR group and 10 in the CCA group)—limits the statistical power and generalizability of our findings. Larger cohorts are needed to validate these observations and draw more robust conclusions. Additionally, some results, such as the differences in alpha diversity, were not statistically significant, likely due to the limited sample size. The cross-sectional design of this study is another limitation, as it prevents conclusions about causality between microbial alterations and the development of CCA. Longitudinal studies are necessary to establish temporal relationships and better understand the role of the microbiota in disease progression. In addition, using individuals with normal bile microbiota as a control group could have provided a valuable reference point for comparing malignant and benign biliary diseases. However, collecting bile samples from healthy individuals presents considerable challenges. The primary obstacles are ethical and safety concerns, as obtaining bile samples typically requires invasive procedures such as an ERCP, percutaneous transhepatic cholangiography, or surgery. These procedures involve significant risks, including infection, bleeding, and pancreatitis, making it ethically unacceptable to expose healthy individuals to such risks solely for research purposes. While we used PICRUSt for functional profiling, the predicted microbial functions were not experimentally validated. Including functional assays in future research would enhance the biological relevance and robustness of these findings. Furthermore, despite strict inclusion criteria, factors such as dietary habits, environmental exposures, or undiagnosed subclinical conditions may have influenced bile microbiota composition, potentially confounding the results. Although 16S rRNA gene sequencing provided valuable insights, its inherent limitations must be considered, as it does not capture the full genomic potential of the microbiota. Whole-genome shotgun sequencing could offer a more comprehensive understanding of the diversity and functional capabilities of the bile microbiota, enabling deeper insights into the microbiome’s role in CCA. These limitations underscore the need for larger, longitudinal studies that include advanced genomic techniques and experimental validations to confirm and expand upon our findings.

## 4. Materials and Methods

### 4.1. Sample Collection

This study enrolled 25 patients diagnosed with gallbladder stones, gallbladder polyps, or CCA. Written informed consent was obtained from all participants for their involvement in our clinical and molecular analyses and for the publication of the study data. The participants were divided into two groups: a CTR group consisting of 15 patients with gallstones (n = 10) or gallbladder polyps (n = 5), who underwent treatment via ERCP or LC, and a CCA group comprising 10 patients diagnosed with CCA. Strict inclusion criteria were applied to minimize potential confounding factors affecting the bile microbiome. Participants were required to have no history of endoscopic sphincterotomy or biliary surgery, no acute cholangitis or cholecystitis at the time of diagnosis, and to have received no antibiotic therapy for three months prior to the procedures. Bile samples (10 mL) were collected from all participants using sterile techniques during an ERCP or LC. For ERCP, samples were obtained with side-viewing endoscopes (TJF240/JF-260V; Olympus, Tokyo, Japan) and sterile sphincterotome catheters to avoid contamination. During LC, bile was aspirated directly from the gallbladder into sterile disposable syringes prior to its removal. All samples were immediately transferred to sterile Falcon 15 mL conical tubes (Corning Inc., New York, NY, USA) and stored at −80 °C to ensure their stability and prevent contamination. This standardized collection and storage process maintained the integrity of the bile samples for subsequent microbiome analyses.

### 4.2. DNA Extraction

Total DNA was extracted from non-centrifuged bile samples using the FastDNA^®^ SPIN Kit for Soil (MP Biomedicals, Santa Ana, CA, USA), following the manufacturer’s protocol. The quantity of the extracted DNA was measured using a Qubit 2.0 Fluorometer (Life Technologies, Carlsbad, CA, USA), and the quality of the DNA was assessed using an E-Gel electrophoresis system (Life Technologies).

### 4.3. Next-Generation Sequencing of 16S Ribosomal RNA Gene

The extracted DNA underwent polymerase chain reaction (PCR) amplification using fusion primers designed specifically to target the V3–V4 regions of the 16S rRNA gene for bacterial identification. The fusion primers employed were 341F (5′-AATGATACGGCGACCACCGAGATCTACACXXXXXXXXCGTCGGCAGCGTCAGATGTGTATAAGAGACAGCCTACGGGNGGCWGCAG-3′) and 805R (5′-CAAGCAGAAGACGGCATACGAGATXXXXXXXXGTCTCGTGGGCTCGGAGATGTGTATAAGAGACAGGACTA-CHVGGGTATCTAATCC-3′), with the target region sequences underlined. These primers were sequentially constructed to include P5 (P7) graft binding sequences, i5 (i7) indices, Nextera consensus sequences, sequencing adapters, and the target region sequences. PCR amplification was performed under the following thermal cycling conditions: an initial denaturation at 95 °C for 3 min; 25 cycles of denaturation at 95 °C for 30 s, annealing at 55 °C for 30 s, and extension at 72 °C for 30 s; and a final elongation at 72 °C for 5 min. The resulting PCR products were purified using CleanPCR (CleanNA, Alphen aan den Rijn, Waddinxveen, The Netherlands) to eliminate non-target products. The quality and integrity of the purified PCR products were assessed using 1% agarose gel electrophoresis and a Bioanalyzer 2100 system (Agilent, Palo Alto, CA, USA) with a DNA 7500 chip. Pooled amplicons were subsequently sequenced as 2 × 250 bp paired-end reads using the MiSeq Sequencing System (Illumina Inc., San Diego, CA, USA) at Chunlab, Inc. (Seoul, Republic of Korea). The sequencing procedure, which targeted the amplified 16S V3–V4 region, was carried out following the manufacturer’s protocols.

### 4.4. Bioinformatics Analysis of Bile Microbiota

The raw sequencing reads underwent quality control to ensure the reliability of the data. Low-quality reads, defined as those with a Phred quality score < Q25, were removed using Trimmomatic version 0.32. Following the filtering process, paired-end sequence data were merged with the fastq_mergepairs command in VSEARCH version 2.13.4, using the default parameters [29]. Primer sequences were then trimmed using Myers and Miller’s alignment algorithm, with a similarity cutoff of 0.8. Non-specific amplicons that did not encode 16S rRNA were identified and removed using nhmmer from the HMMER software package version 3.2.1, utilizing HMM profiles [30]. To streamline the dataset, unique reads were extracted and redundant reads were clustered using the derep_fulllength command in VSEARCH [29]. This workflow ensured that only high-quality, relevant sequences were retained for further analysis.

### 4.5. Taxonomic Profiling Analysis

The EzBioCloud Apps 16S-based microbial taxonomic profiling (MTP) platform (ChunLab, Inc., Seoul, Republic of Korea, https://www.ezbiocloud.net/; accessed on 23 August 2023) was employed to investigate metagenomic variations in bile microbial communities. Taxonomic assignments were performed using the EzBioCloud 16S rRNA database [31], with precise pairwise alignment to enhance accuracy [29]. Chimeric reads were identified and removed using the UCHIME algorithm, and sequences with less than 97% similarity were filtered out [32], ensuring that only non-chimeric sequences were retained for further analysis. Operational taxonomic units (OTUs) were clustered at 97% sequence identity, and representative sequences from each OTU were taxonomically classified based on the EzBioCloud database [33]. Reads that could not be assigned to a species with ≥97% similarity underwent de novo clustering using the cluster_fast command in VSEARCH to generate additional OTUs [29]. OTUs consisting of single reads were excluded to improve the reliability of the analysis. Taxonomic profiling was performed for each sample, and comparative analyses of microbial communities were conducted using the comparative MTP analyzer within EzBioCloud Apps (https://www.ezbiocloud.net/apps/; accessed on 23 August 2023).

For the diversity analysis, read counts were normalized through random subsampling, and alpha diversity indices were calculated using Mothur (https://www.mothur.org/; accessed on 23 August 2023) [34]. These indices included Simpson, Shannon, NPShannon, Jackknife, Chao, ACE, and phylogenetic diversity, alongside rarefaction and rank abundance curves. A significance threshold of 0.05 and an effect size of 3 were applied for diversity assessments. Beta diversity distances, reflecting variations in microbial community composition, were calculated using algorithms such as Jensen–Shannon, Generalized UniFrac, Fast UniFrac, and Bray–Curtis. A PCoAwas performed using the comparative MTP analyzer, enabling a visualization of microbiota composition differences, with PCoA plots facilitating comparisons between samples [35]. To identify taxa that significantly differed between groups, an LEfSe analysis was conducted, using both statistical significance and biological relevance to identify potential biomarkers. The functional profiling of microbial communities was predicted using PICRUSt [36] and MinPath [37], offering insights into the functional differences between microbial groups [38].

### 4.6. Statistical Analysis

The metagenomic differences in bile microbial communities between the two groups were analyzed using the statistical methods implemented in R software (version 3.1.2, R Foundation for Statistical Computing, Vienna, Austria). To compare microbial compositions, the Kruskal–Wallis test, Mann–Whitney U test, and Wilcoxon rank-sum test were employed. A significance level of *p* < 0.05 was applied to all analyses to establish statistical significance.

## 5. Conclusions

This study offers valuable insights into the composition of the bile microbiota and its association with CCA. It highlights potential microbial biomarkers and structural differences between benign and malignant diseases. However, further research with larger cohorts and robust study designs is essential to validate and expand upon these findings. Understanding the distinct bile microbiota profiles linked to CCA presents promising opportunities for the development of non-invasive diagnostic biomarkers. Additionally, therapeutic strategies targeting the modulation of the bile microbiota could serve as adjunctive treatments to inhibit tumor progression. Future studies should focus on expanding participant cohorts, employing longitudinal designs, and integrating multi-omics approaches to enhance the reliability and translational potential of these findings.

## Data Availability

Data are contained within the article.
